# Limitation of diagnostic value of cone-beam CT in detecting apical root isthmuses

**DOI:** 10.1590/1678-7757-2019-0168

**Published:** 2020-03-27

**Authors:** Elen de Souza TOLENTINO, Pablo Andrés AMOROSO-SILVA, Murilo Priori ALCALDE, Heitor Marques HONÓRIO, Lilian Cristina Vessoni IWAKI, Izabel Regina Fischer RUBIRA-BULLEN, Marco Antônio HÚNGARO-DUARTE

**Affiliations:** 1 Universidade Estadual de Maringá Departamento de Odontologia MaringáParaná Brasil Universidade Estadual de Maringá , Departamento de Odontologia , Maringá , Paraná , Brasil .; 2 Universidade Estadual de Londrina Departamento de Odontologia Restauradora LondrinaParaná Brasil Universidade Estadual de Londrina , Departamento de Odontologia Restauradora , Londrina , Paraná , Brasil .; 3 Universidade de São Paulo Faculdade de Odontologia de Bauru Departamento de Dentística, Endodontia e Materiais Odontológicos BauruSão Paulo Brasil Universidade de São Paulo , Faculdade de Odontologia de Bauru , Departamento de Dentística, Endodontia e Materiais Odontológicos , Bauru , São Paulo , Brasil .; 4 Universidade de São Paulo Faculdade de Odontologia de Bauru Departamento de Odontopediatria, Ortodontia e Saúde Coletiva BauruSão Paulo Brasil Universidade de São Paulo , Faculdade de Odontologia de Bauru , Departamento de Odontopediatria, Ortodontia e Saúde Coletiva , Bauru , São Paulo , Brasil .; 5 Universidade de São Paulo Faculdade de Odontologia de Bauru Departamento de Cirurgia, Estomatologia, Patologia e Radiologia BauruSão Paulo Brasil Universidade de São Paulo , Faculdade de Odontologia de Bauru , Departamento de Cirurgia, Estomatologia, Patologia e Radiologia , Bauru , São Paulo , Brasil .

**Keywords:** Anatomy, Cone-beam computed tomography, Endodontics, Microcomputed tomography, Root canal

## Abstract

Isthmuses are reported as common anatomic complexities in teeth often associated with failures in endodontic treatment. They should be considered before starting treatment and a preoperative computed tomography scan (CT) may demonstrate these complexities.

**Objective:**

To assess the diagnostic value of the highest resolution settings of a cone-beam CT (CBCT) system in identifying and measuring apical isthmuses, using micro-CT as reference.

**Methodology:**

After micro-CT scanning, 40 humans’ lower first molars with isthmuses in the apical-3 mm of mesial roots were scanned by the highest resolution settings of the New Generation i-Cat ^®^ CBCT equipment. Two blinded observers recorded the detection of isthmuses in CBCT scans. The lengths of isthmuses were compared between micro-CT and CBCT to assess the diagnostic value of CBCT. Quantitative data for sensitivity were represented as percentages (95% confidence interval). The Bland-Altman method was used to assess differences between gold standard lengths (micro-CT) and CBCT lengths.

**Results:**

BCT demonstrated 30 positive findings, representing sensitivity for isthmus identification of 75% (95% CI=0.4114–1.1364). Differences between the lengths in micro-CT (1.99±0.40 mm) and CBCT (1.53±0.41 mm) were significant (p<0.0001).

**Conclusion:**

The CBCT device used presented limited diagnostic value in the identification and measurement of apical isthmuses in the mesial roots of lower molars. In some cases, the actual anatomy of the apical root canal may not be completely delineated in this type of CBCT system, even using the highest resolution settings.

## Introduction

Isthmuses, defined as ribbon-shaped communications between the mesiobuccal and mesiolingual canals containing pulp tissue, ^1 , 2^ are reported as common anatomic complexities in the posterior teeth, which cannot be detected in radiographs. These regions may work as reservoirs of microorganisms ^3^ and their insufficient cleaning may result in endodontic treatment failure. The highest frequencies of isthmuses in human permanent teeth are found in the lower first molars, ^4^ and its prevalence is higher at the apical level, ^5 - 8^ considered as a critical area. ^2^ An association between apical isthmuses and apical periodontitis has been reported. ^9 , 10^ Thus, their detection may be important to improve the predictability of treatment.

Micro computed tomography (micro-CT) is currently considered the gold standard technology for identifying isthmuses in teeth *ex vivo* , ^6^ since it allows an in-depth study of root canal anatomy without destroying the samples. ^2^ For clinical practice, cone-beam CT (CBCT) is an available alternative as it provides three-dimensional and multiplanar analysis with reduced radiation doses when compared with fan-beam CT. ^11^ Nowadays, several CBCT systems are available and some parameters could interfere in the quality of the exam, such as the slice thickness, spatial resolution, detector design, the field of view (FOV) and voxel size. It is known that small FOV and voxels provide higher spatial resolutions. ^12^ However, studies using CBCT to detect and to describe isthmuses of root canal systems are scarce, ^4 , 7 , 13^ and the influence of the different settings in the image quality is little approached.

As the isthmuses play an important role in root canal treatment, this study aimed to assess the diagnostic value of the highest resolution settings of a CBCT system to identify and to measure apical isthmuses of lower molars root canals, using micro-CT scanning as the reference method.

## Material and Methods

After approval by the Research Ethics Committee (REC #1.929.037 in compliance with the Helsinki Declaration) and sample calculation—performed using repeated analysis of variance (ANOVA), the measures with a significance level of 5% and power of analysis of 95%—, 40 lower first molars (extracted for different reasons and stored in 10% neutral-buffered formalin solution) with type II ^14^ isthmuses in the apical third of mesial roots were selected. Molars with calcified root canals, incomplete rhizogenesis, resorption, root fractures and/or endodontic treatment were excluded, and both sex and age of the patients were unknown ^13^ . The teeth were imaged by micro-CT scanning (SkyScan 1174v2, Bruker Corporation, Kontich, Antwerp, Belgium); (50 kV, 800 mA, 360°, 19.6 μm isotropic resolution). CTVol v.2.2.1 and data viewing software (Bruker-microCT) were used to adequate the images for visualization and qualitative evaluation of the isthmuses.

**Figure 1:**
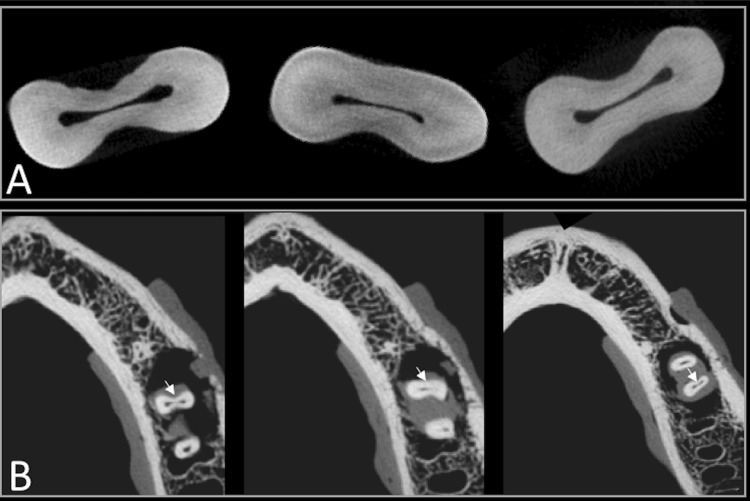
A. Type II isthmuses (definite connections between the two main canals in the mesial root of lower first molars) at the apical level in micro-CT images; B. The same apical isthmuses detected in the CBCT images

**Figure 2:**
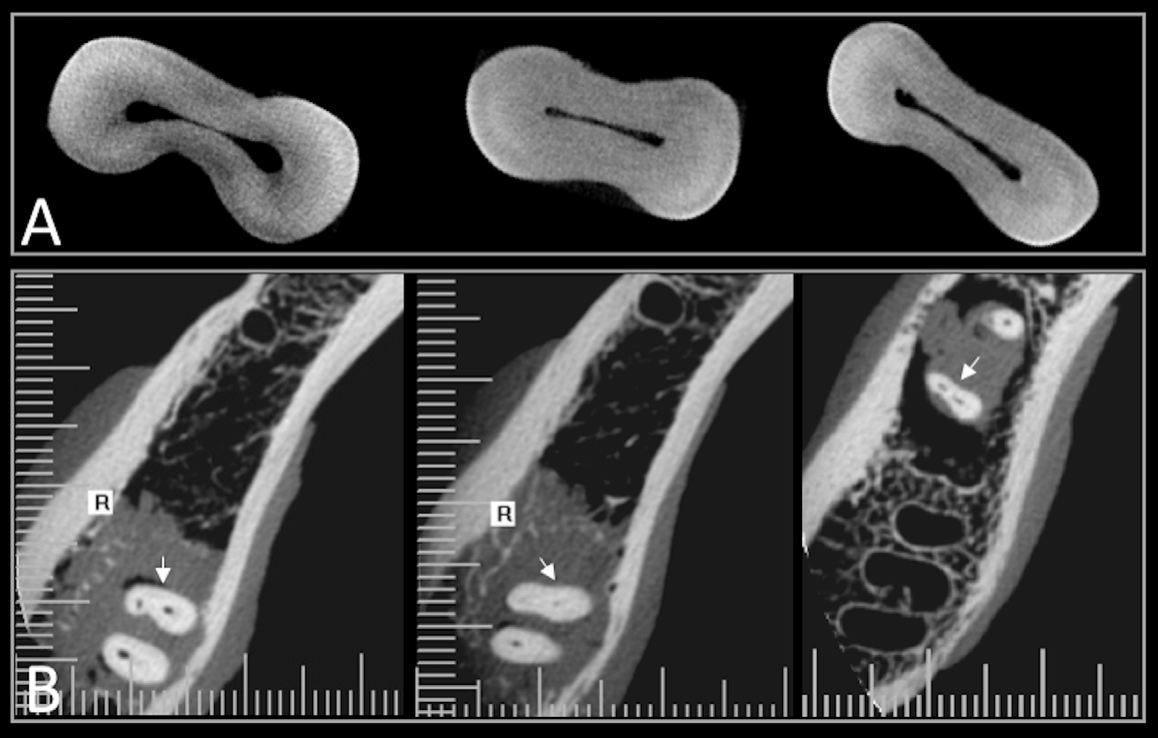
A. Type II isthmuses at the apical level in micro-CT images; B. The same apical isthmuses not detected in the CBCT images

For each mesial root, the isthmus was located by visualizing the apical 3-mm axial images ( Figures 1A and 2A ). Each specimen had its length registered, at the greatest thickness region of the isthmus in the apical 3-mm. ^13^ The first millimeter (from the radiographic apex to the cementum-dentin junction level) was not considered, i.e., the apical-3 mm from the ideal working length was assessed.

To simulate the radiographic appearance of the periodontal space, the teeth were uniformly covered with a thin layer of utility wax Tenatex Red (Kemdent▢, Swindon, Wiltshire, England) and fixed in preformed sockets in dry human mandibles ^15^ coated with three layers of wax to provide some level of soft tissue simulation (buccally and lingually). ^13^ Then, each tooth was scanned individually in the New Generation i-Cat ^®^ (Imaging Science International, Hatfield, Pennsylvania, United States), following the manufacturer’s protocol. The highest resolution settings of the equipment were used (8x8 cm FOV, 125 μm voxel size, 120 Kv, 5 mA), and the images were evaluated using the scanner own software (Xoran 3.1.62 version, Xoran Technologies LLC, Ann Arbor, Michigan, United States).

Two independent and external examiners (radiologists with more than 10 years of experience with CBCT) performed a blind analysis of the apical 1- to 3-mm level in the CBCT scans and registered the presence or absence of the isthmus in each tooth. Zoom, filter and contrast tools could be used to simulate routine reality. The presence of isthmuses was analyzed using a previous described map-reading strategy, ^4 , 13^ with axial scanning of 0.125 mm/0.125-mm slices moving from the pulp orifice to the root apex to evaluate the apical third. Length measurements of isthmuses in micro-CT and CBCT scans were performed by a single examiner (ten exams were randomly selected for replicate measurements within 15 days).

Inter-observer concordance reliability for isthmus detection or non-detection were assessed using the Kappa index. To assess intra-examiner concordance for length measurements, the intraclass correlation coefficient (ICC) was used. Results of diagnostic CBCT scans were reported for the detection of isthmuses, considering micro-CT as the reference standard. 95% confidence interval (CI) was used to report the sensitivity estimated for CBCT. The Bland-Altman method was used to evaluate differences between gold standard lengths (micro-CT) and CBCT lengths. Data were tested with SPSS 15.0 (SPSS Inc., Chicago, Illinois, United States) with a significance level of 5% (α=0.05). The Bland-Altman graph was performed at MedCalc ^®^ (MedCalc Software bvba, Ostend, West Flanders, Belgium).

## Results

Kappa value for inter-observer concordance was perfect (1.00). Intra-examiner agreement was excellent for length measurements (ICC=0.94 and 95%CI: 0.87–98). The detection and non-detection of isthmuses in CBCT scans are shown in Figures 1B and 2B , respectively. A total of 30 positive findings indicated sensitivity for isthmus identification in 75% (95% CI=0.4114–1.1364) (p<0.001) compared with the micro-CT were found.

**Figure 3:**
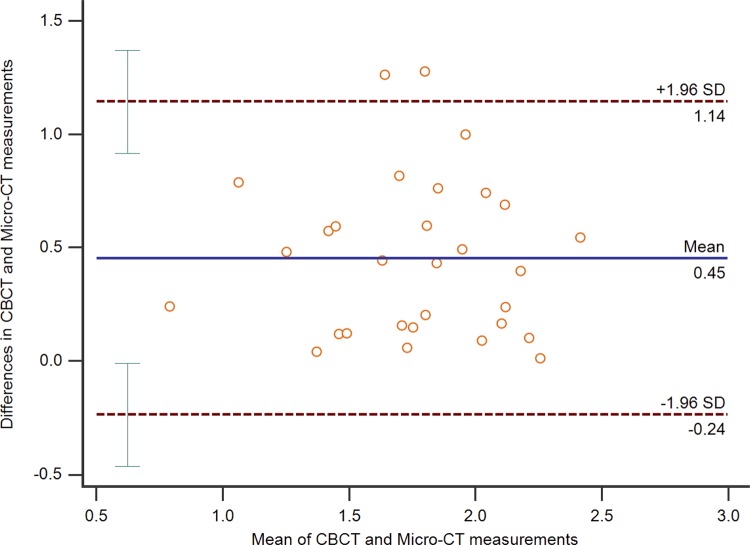
Bland-Altman graph of length measurements (mm) of isthmuses. The blue line represents the mean difference (0.45 mm) between the micro-CT and CBCT measurements. The dotted lines correspond to the limits of concordance (mean difference±1.96 standard deviations)

The CBCT images showed the length of the isthmuses in the apical 3-mm varied from 0.67 mm to 2.25 mm with a mean value of 1.53±0.41 mm. The mean value of the micro-CT isthmus lengths ranged between 0.91 mm and 2.68 mm with a mean value of 1.99±0.40 mm ( Table 1 ). The length of the isthmuses could be measured in the CBCT protocol in 75% of the sample (n=30), with significant differences when compared with the micro-CT (p<0.0001, mean=0.776±0.657). For length measurements, the differences between micro-CT and CBCT ranged between 0.012 mm and 1.27 mm (mean difference of 0.45 mm) ( Figure 3 ). Regression analysis showed no proportional bias.

**Table 1:** Measurement of the isthmuses length values in micro-TC and CBCT, differences and means of both methods (mm)

Micro-CT	CBCT	Differences	Means
1.457	0.67	0.787	1.06
0.911	0.67	0.241	0.79
1.492	1.01	0.482	1.25
2.272	1.01	1.262	1.64
1.704	1.13	0.574	1.41
1.743	1.15	0.593	1.44
2.438	1.16	1.278	1.79
2.106	1.29	0.816	1.69
1.392	1.35	0.042	1.37
1.519	1.4	0.119	1.45
1.853	1.41	0.443	1.63
1.551	1.43	0.121	1.49
2.46	1.46	1	1.96
2.233	1.47	0.763	1.85
2.106	1.51	0.596	1.8
1.786	1.63	0.156	1.7
2.061	1.63	0.431	1.84
2.411	1.67	0.741	2.04
1.827	1.68	0.147	1.75
2.193	1.7	0.493	1.94
1.759	1.7	0.059	1.72
1.903	1.7	0.203	1.8
2.461	1.77	0.691	2.11
2.378	1.98	0.398	2.1
2.07	1.98	0.090	2.01
2.239	2	0.239	2.11
2.187	2.02	0.167	2.1
2.686	2.14	0.546	2.4
2.262	2.16	0.102	2.21
2.262	2.25	0.012	2.25

## Discussion

The presence of anatomical complexities such as isthmuses is a key challenge in endodontic therapy of posterior teeth. ^1^ Mesial roots of lower molars have a high prevalence of these modifications, ^6^ which are often associated with failure of endodontic treatment. ^2 , 3^ The apical level is the critical area ^2^ and its incomplete cleaning is responsible for most cases of failure in endodontic treatment. ^9^ In the cervical third, the detection of isthmuses in CBCT exams is easy due to its greater thickness, area and length, and access by rotatory or ultrasound instruments is easier. ^16^ However, when the isthmuses begin in the apical third, the access to these areas is hampered. Despite the continuous progress in endodontic treatment, cleaning and disinfecting isthmuses is still a clinical challenge. ^16^

In this study, micro-CT scanning was used as a reference, since it provides high quality and detailed images of the root canal anatomy without destroying the sample. ^17^ In the clinical routine, this technology cannot be used and CBCT images are being increasingly sought-after by endodontists. This three-dimensional method eliminates artifacts resulting from superimposition of adjacent structures, providing a dynamic visualization not achieved on radiographs. ^4^

The Next Generation i-Cat CBCT unit was used in this study for its ability to offer such different acquisition settings, supplying the demands of many specialties. Furthermore, this system is one of the most used units in the world. ^18^ The i-Cat system is considered a large volume device with several available FOVs, and the smallest FOV (8x8 cm) combined with the smallest voxel size available (0.125 mm) was used in this study. It is known that larger FOV selection provides less spatial resolution and higher amount of X-ray noise. ^11 , 19^ Similarly, the voxel size also influences image resolution, ^19^ with smaller voxels providing better images, despite increasing the scanning and reconstruction time. ^20^ In Endodontics, where high resolution images are often necessary, small FOV and voxels are preferred.

Despite the used CBCT unit is a flat-panel detector—based system (FPD) reported as better than image intensifier tube/charged coupled device combination (IIT/CCD) detectors in terms of spatial resolution and noise—a sensitivity for apical isthmus detection of 75% was found, with a relatively high rate of false negatives, not enabling fully reliable identification of these alterations within teeth, even though the highest resolution settings were used. In other words, CBCT failed in identifying some apical isthmuses, demonstrating its restriction and potential for underdiagnosis in this task. The expectation was that micro-CT and CBCT would not produce equable values for isthmuses measurements because micro-CT has a smaller voxel size and better image quality. ^13^ Although an average difference of 0.45 mm between both methods in length measurement may be clinically irrelevant, more reliable results could be achieved for CBCT higher protocol measurements and, therefore, to associate these values to a better clinical outcome. Moreover, in Endodontics, even sub-millimetric distances can represent important reservoirs for microorganisms, hampering the success of an endodontic treatment.

Other image quality parameters are more likely to influence the visualization of isthmuses. Among those parameters, spatial resolution, scatter effects, partial volume phenomenon and presence of artifacts might be considered, which may have influenced the sensitivity results. Some authors ^21 , 22^ report that the CBCT spatial resolution is relevant in the image quality due to the high accuracy required to exam small structures (i.e., isthmuses). The image quality could be reduced by the presence of artifacts, scatter effects, patient’s movement in magnitudes greater than voxel size and other factors. ^22^ Furthermore, the partial volume effect—which occurs when the scan voxel resolution exceeds the spatial resolution of the structure being analyzed— ^22^ also limits the CBCT ability in identifying thinner structures. The CBCT protocol of 0.125 mm as voxel size is probably larger than the diameter or the length of the isthmuses. Therefore, the examiners could not visually detect the terminal portion of the isthmuses. As a consequence of the lack of details provided by the highest resolution combination (8x8 cm; 0.125 mm) of the machine, the images were not good enough to treat the apical region of the teeth. Clinically, residual bacteria are more abundant and frequent in isthmuses and they are never completely cleaned nor obturated, ^10 , 23^ being important in cases of endodontic treatment failure. In this context, CBCT diagnostic value should not be overestimated ^22^ and the presence of these anastomosis should be considered whether identified in CBCT or not. ^13^

As limitation of this study, only one type of isthmus was evaluated. Furthermore, the methodology could not address some factors of clinical routine, such as the presence of pins, posts and root canal fillings, moisture, patient’s movement, and other sources of artifacts. One hypothesis is that the sensitivity of CBCT is probably lower in clinical practice because of these factors. However, the literature does not show significant differences when different methodologies are used. ^1 , 7^

The variability of human tooth anatomy should be considered before any endodontic treatment and a preoperative CBCT scan is extremely important in most cases. However, the higher radiation dose of CBCT compared with conventional radiographs limits the use of it in selected cases, always after a meticulous clinical examination. These results show the CBCT diagnostic value to identify isthmuses in the apical third was limited; therefore, an exam for this sole objective should not be indicated. As we demonstrated, CBCT may underdiagnoses these apical variations in some cases, which may affect the outcome of root canal treatment. Even when not detected by CBCT, an isthmus may be present and failure in its identification may influence the endodontist, who can understand the anatomy of the root canal as simple, thus performing insufficient cleaning. ^13^ During root canal treatment, the presence of isthmuses may affect effective debridement. ^24^ The accumulation of dentine debris in these areas could harbor bacteria and serve as a nest for reinfection and treatment failure. ^25^ When treating lower first molars, especially mesial root canals, thorough irrigation should be considered as a routine procedure. ^24^ This procedure is crucial for improved cleaning and disinfection of the whole root canal system.

## Conclusion

Despite using the high-resolution settings, the CBCT images had limited sensibility (75%) to detect apical isthmuses in mesial roots of lower first molars and to measure their lengths. Clinicians should be aware that what they are looking at in CBCT images may not be the actual anatomy of the apical region in some cases.

## References

[1] - Vertucci FJ . Root canal morphology and its relationship to endodontic procedures . Endod Topics . 2005 ; 10 ( 1 ): 3 - 29 . doi: 10.1111/j.1601-1546.2005.00129.x

[2] - Paqué F , Laib A , Gautschi H , Zehnder M . Hard-tissue debris accumulation analysis by high-resolution computed tomography scans . J Endod . 2009 ; 35 ( 7 ): 1044 - 7 . doi: 10.1016/j.joen.2009.04.026 10.1016/j.joen.2009.04.026 19567331

[3] - Carr GB , Schwartz RS , Schaudinn C , Gorur A , Costerton JW . Ultrastructural examination of failed molar retreatment with secondary apical periodontitis: an examination of endodontic biofilms in an endodontic retreatment failure . J Endod . 2009 ; 35 ( 9 ): 1303 - 9 . doi: 10.1016/j.joen.2009.05.035 10.1016/j.joen.2009.05.035 19720237

[4] - Estrela C , Rabelo LE , Souza JB , Alencar AH , Estrela CR , Sousa Neto MD , et al . Frequency of root canal isthmi in human permanent teeth determined by cone-beam computed tomography . J Endod . 2015 ; 41 ( 9 ): 1535 - 9 . doi: 10.1016/j.joen.2015.05.01 10.1016/j.joen.2015.05.016 26187423

[5] - Mannocci F , Peru M , Sherriff M , Cook R , Pitt Ford TR . The isthmuses of the mesial root of mandibular molars: a micro-computed tomographic study . Int Endod J . 2005 ; 38 ( 8 ): 558 - 63 . 10.1111/j.1365-2591.2005.00994.x 16011775

[6] - Gu L , Wei X , Ling J , Huang X . A microcomputed tomographic study of canal isthmuses in the mesial root of mandibular first molars in a Chinese population . J Endod . 2009 ; 35 ( 3 ): 353 - 6 . doi: 10.1016/j.joen.2008.11.029 10.1016/j.joen.2008.11.029 19249594

[7] 7 - Tahmasbi M , Jalali P , Nair MK , Barghan S , Nair UP . Prevalence of middle mesial canals and isthmi in the mesial root of mandibular molars: an *in vivo* cone-beam computed tomographic study . J Endod . 2017 ; 43 ( 7 ): 1080 - 3 . doi: 10.1016/j.joen.2017.02.008 10.1016/j.joen.2017.02.008 28527840

[8] - Ordinola-Zapata R , Martins JN , Niemczyk S , Bramante CM . Apical root canal anatomy in the mesiobuccal root of maxillary first molars: influence of root apical shape and prevalence of apical foramina - a micro-CT study . Int Endod J . 2019 ; 52 ( 8 ): 1218 - 27 . doi: 10.1111/iej.13109. 10.1111/iej.13109 30849181

[9] - Ricucci D , Siqueira JF Jr . Biofilms and apical periodontitis: study of prevalence and association with clinical and histopathologic findings . J Endod . 2010 ; 36 ( 8 ): 1277 - 88 . doi: 10.1016/j.joen.2010.04.007 10.1016/j.joen.2010.04.007 20647081

[10] - Siqueira JF Jr , Perez AR , Marceliano-Alves MF , Provenzano JC , Silva SG , Pire , FR et al . What happens to unprepared root canal walls: a correlative analysis using micro-computed tomography and histology/scanning electron microscopy . Int Endod J . 2018 ; 51 ( 5 ): 501 - 8 . doi: 10.1111/iej.12753 10.1111/iej.12753 28196289

[11] - Hassan BA , Payam J , Juyanda B , van der Stelt P , Wesselink PR . Influence of scan setting selections on root canal visibility with cone beam CT . Dentomaxillofac Radiol . 2012 ; 41 ( 8 ): 645 - 8 . doi: 10.1259/dmfr/27670911 10.1259/dmfr/27670911 PMC3528194 23166361

[12] - Kamburoglu K , Onder B , Murat S , Avsever H , Yüksel S , Paksoy CS . Radiographic detection of artificially created horizontal root fracture using different cone beam CT units with small fields of view . Dentomaxillofac Radiol . 2013 ; 42 ( 4 ): 20120261 . doi: 10.1259/dmfr.20120261 10.1259/dmfr.20120261 PMC3667510 23420851

[13] - Tolentino ES , Amoroso-Silva PA , Alcalde MP , Honório HM , Iwaki LC , Rubira-Bullen IR , et al . Accuracy of high-resolution small-volume cone-beam computed tomography in detecting complex anatomy of the apical isthmi: ex vivo analysis . J Endod . 2018 ; 44 ( 12 ): 1862 - 6 . doi: 10.1016/j.joen.2018.08.015 10.1016/j.joen.2018.08.015 30390974

[14] - Hsu YY , Kim S . The resected root surface. The issue of canal isthmuses . Dent Clin North Am . 1997 ; 41 ( 3 ): 529 - 40 . 9248689

[15] - Hassan B , Metska ME , Ozok AR , van der Stelt P , Wesselink PR . Comparison of five cone beam computed tomography systems for the detection of vertical root fractures . J Endod . 2010 ; 36 ( 1 ): 126 - 9 . doi: 10.1016/j.joen.2009.09.013 10.1016/j.joen.2009.09.013 20003950

[16] - Susin L , Liu Y , Yoon JC , Parente JM , Loushine RJ , Ricucci D , et al . Canal and isthmus debridement efficacies of two irrigant agitation techniques in a closed system . Int Endod J . 2010 ; 43 ( 12 ): 1077 - 90 . doi: 10.1111/j.1365-2591.2010.01778.x 10.1111/j.1365-2591.2010.01778.x PMC2970719 20726910

[17] - Amoroso-Silva PA , Ordinola-Zapata R , Duarte MA , Gutmann JL , del Carpio-Perochena A , Bramante CM , et al . Micro-computed tomographic analysis of mandibular second molars with C-shaped root canals . J Endod . 2015 ; 41 ( 6 ): 890 - 5 . doi: 10.1016/j.joen.2015.01.021 10.1016/j.joen.2015.01.021 25732399

[18] - Van Dessel J , Huang Y , Depypere M , Rubira-Bullen I , Maes F , Jacobs R . A comparative evaluation of cone beam CT and micro-CT on trabecular bone structures in the human mandible . Dentomaxillofac Radiol . 2013 ; 42 ( 8 ): 20130145 . doi: 10.1259/dmfr.20130145 10.1259/dmfr.20130145 PMC3922263 23833320

[19] 19 - Katsumata A , Hirukawa A , Okumura S , Naitoh M , Fujishita M , Ariji E , et al . Relationship between density variability and imaging volume size in cone-beam computerized tomographic scanning of the maxillofacial region: an *in vitro* study . Oral Surg Oral Med Oral Pathol Oral Radiol Endod . 2009 ; 107 ( 3 ): 420 - 5 . doi: 10.1016/j.tripleo.2008.05.049 10.1016/j.tripleo.2008.05.049 18715805

[20] - Bagis N , Eren H , Kolsuz ME , Kurt MH , Avsever H , Orhan K . Comparison of the burr and chemically induced periodontal defects using different field-of-view sizes and voxel resolutions . Oral Surg Oral Med Oral Pathol Oral Radiol . 2018 ; 125 ( 3 ): 260 - 7 . doi: 10.1016/j.oooo.2017.11.010 10.1016/j.oooo.2017.11.010 29273196

[21] - Bamba J , Araki K , Endo A , Okano T . Image quality assessment of three cone beam CT machines using the SEDENTEXCT CT phantom . Dentomaxillofac Radiol . 2013 ; 42 ( 8 ): 20120445 . doi: 10.1259/dmfr.20120445 10.1259/dmfr.20120445 PMC3922264 23956235

[22] - Brüllmann D , Schulze RKW . Spatial resolution in CBCT machines for dental/maxillofacial applications — what do we know today? Dentomaxillofac Radiol . 2015 ; 44 ( 1 ): 20140204 . doi: 10.1259/dmfr.20140204 10.1259/dmfr.20140204 PMC4614158 25168812

[23] - Vera J , Siqueira JF Jr , Ricucci D , Loghin S , Fernández N , Flores B , et al . One- versus two-visit endodontic treatment of teeth with apical periodontitis: a histobacteriologic study . J Endod . 2012 ; 38 ( 8 ): 1040 - 52 . doi: 10.1016/j.joen.2012.04.010 10.1016/j.joen.2012.04.010 22794203

[24] 24 - Hu X , Huang Z , Huang Z , Lei L , Cui M , Zhang X . Presence of isthmi in mandibular mesial roots and associated factors: an *in vivo* analysis . Surg Radiol Anat , 2019 ; 41 : 815 - 22 . doi: 10.1007/s00276-019-02231-w 10.1007/s00276-019-02231-w PMC6570693 30937566

[25] - Leoni GB , Versiani MA , Silva-Sousa YT , Bruniera JF , Pécora JD , Sousa-Neto MD . Ex vivo evaluation of four final irrigation protocols on the removal of hard-tissue debris from the mesial root canal system of mandibular first molars . Int Endod J . 2017 ; 50 ( 4 ): 398 - 406 . doi: 10.1111/iej.12630 10.1111/iej.12630 26992452

